# A simple and flexible approach for detecting small numbers of SNPs

**DOI:** 10.3389/fpls.2025.1748099

**Published:** 2025-12-19

**Authors:** Renbo Yu, Jie Liu, Yu Niu, Xu Han, Xiaoyi Wang, Yan Yang

**Affiliations:** 1 Tropical Crops Genetic Resources Institute, State Key Laboratory of Tropical Crop Breeding, Chinese Academy of Tropical Agricultural Sciences, Haikou, Hainan, China; 2 Sanya Research Institute, Chinese Academy of Tropical Agricultural Sciences, Sanya, Hainan, China; 3 Key Laboratory of Crop Gene Resources and Germplasm Enhancement in Southern China, Ministry of Agriculture and Rural Affairs, Haikou, China; 4 Key Laboratory of Tropical Crops Germplasm Resources Genetic Improvement and Innovation of Hainan Province, Haikou, China; 5 Rubber Research Institute, Chinese Academy of Tropical Agricultural Sciences, Haikou, Hainan, China

**Keywords:** detection methods, forward primer, mismatch, single nucleotide polymorphism (SNP), stress resistance

## Abstract

SNP markers represent the most extensively distributed and abundant type of polymorphic markers. Although researchers have developed high-throughput detection methods for SNP markers, there remains a lack of simple, flexible, and cost-effective approaches for detecting small numbers of SNPs. To address this need, this study proposes a method for detecting SNPs based on T-C mismatch. Specifically, two upstream primers were designed with a single base difference at the 3’ end. Additionally, a T-C mismatch was introduced within 5 bp upstream of the 3’ end to distinguish SNPs. Detection was achieved through PCR amplification and subsequent gel electrophoresis analysis. In this study, twelve random SNPs from bitter gourd were selected, and the detection efficiency was found to be 83.3%. The proposed SNP detection method is characterized by its simplicity and flexibility, offering an effective tool for molecular marker-assisted selection breeding in applications such as stress resistance improvement and agricultural productivity enhancement.

## Introduction

Molecular markers, discovered and applied since 1980s, such as random amplified polymorphic DNA (RAPD) (Tingey and del Tufo, 1993), simple sequence repeat (SSR) (Zietkiewicz et al., 1994), sequence characterized amplified region (SCAR) (Deng et al., 1997), cleaved amplified polymorphic sequence (CAPS) (Konieczny and Ausubel, 1993), and amplified fragment length polymorphism (AFLP) (Mueller and Wolfenbarger, 1999), played significant roles in marker assisted crop breeding and human disease diagnosis during the past few decades (Huang et al., 1992; Amiteye, 2021). Single nucleotide polymorphisms (SNPs), including transition, transversion, insertion and deletion, employed as a new generation marker in marker assisted selection breeding in recent years. Compared to traditional molecular markers, SNPs are widely distributed in the genome about one SNP per approximately 1000 base pairs in human (Zhou et al., 2001; Koirala et al., 2011) and high throughput SNP detection platforms are available for SNP genotyping.

SNP represents the most prevalent form of genetic variation observed among individuals. The correlation between SNPs and an individual’s response to phenotypic variations, pathogens, and gene functions underscores the critical importance of rapid, sensitive, and reliable methods on SNP detection (Ganal et al., 2009). The detection of SNPs can be achieved through PCR or Sanger sequencing for low throughput, or via array-based detection techniques for high throughput. The field of SNP scoring has witnessed the development of a diverse array of techniques, each possessing distinct advantages and limitations. The Affymetrix arrays possess an exceptionally high probe density, allowing for the incorporation of millions of probes on a single chip. For instance, the Affymetrix Human SNP Array 6.0 encompasses probes targeting 906,600 SNPs (Kanthaswamy et al., 2013). The Illumina Infinium whole-genome genotyping (WGG) technology utilizes a primer extension reaction to assay SNPs, which can be combined with high-density BeadChips to create array platforms capable of genotyping over 1,000,000 SNPs per slide (Gunderson, 2009; Adler et al., 2013). KASP is an allele-specific oligo extension-based PCR assay that utilizes fluorescence resonance energy transfer (FRET) for the detection of genetic variations, such as SNPs and insertions/deletions (InDels), in 96-, 384-, and 1,536-well microtiter plate formats (He et al., 2014).

It could be argued that no single SNP genotyping platform is sufficiently comprehensive to cater to all objectives, the inclusion of certain low-throughput detection methods is also imperative. A multiplex SNP detection system utilizing thin-film optical biosensor silicon chips, arrayed with aldehyde-labeled oligonucleotides and hybridized with PCR amplicons in the presence of a mixture of biotinylated detector probes, exhibits high sensitivity and specificity through color changes on the chip surface (Zhong et al., 2003; Bai et al., 2007). However, the immobilization of nucleotides onto the chip entails significant costs. Another SNP detection approach is based on specific primer extension reactions coupled with PPi detection, and this method has successfully employed DNA primers that contain mismatched bases in the vicinity of their 3’-termini to diminish false positive signals in selective primer extension reactions (Zhou et al., 2001). Nevertheless, this method is technically complex and requires a high level of expertise. A more convenient single nucleotide polymorphism (SNP) detection method, developed by introducing an artificial mismatched base pair into the forward primer and implemented via PCR coupled with agarose gel electrophoresis, has also proven effective (Chen and Schedl, 2021). Although this approach offers operational simplicity, it does not determine which specific base pair configuration yields optimal amplification efficiency. Therefore, there remains a need for a convenient, flexible, effective, and cost-efficient SNP detection method suitable for routine laboratory applications.

Constitutive Photomorphogenic 1 (COP1) and Elongated Hypocotyl 5 (HY5) are master regulatory hub in plants, central not just to light signaling but also to integrating a wide array of stress responses (Chattopadhyay et al., 1998; Lau and Deng, 2012; Han et al., 2020, Xiao et al., 2021, Mankotia et al., 2024). To facilitate the identification of the mutants *cop1–6* and *hy5-215*, we developed a simplified method as an alternative to Sanger sequencing. In this study we found the substitution of the base T with C within a 5 bp region upstream of the SNP significantly enhances primer specificity. In brief, we constructed a simple and low throughput SNP detection system via adding artificial mismatch bases to the forward primer, and this approach is a flexible, rapid, sensitive, and inexpensive way for low throughput SNP scoring.

## Materials and methods

The *Arabidopsis thaliana* wild-type plants utilized in this investigation were of the Columbia ecotype (Col), the *Arabidopsis* mutant of *cop1-6* (McNellis et al., 1994) are in Col background. The seeds were surface-sterilized by immersion in a 10% NaClO solution for 10 minutes and subsequently rinsed with sterile distilled ddH_2_O four times. Following this, the seeds were stored in darkness at a temperature of 4 °C for a period of four days. Subsequently, they were plated on MS medium (4.4 g/L MS, 1% sucrose, 0.8% agar, pH5.8) and transferred to light chambers maintained at a temperature of 22 °C under an intensity of 20,000 lux. Once germinated, the seedlings were transplanted into small pots and cultivated in a greenhouse maintained at 22 °C with a light intensity of 20,000 lux, under long-day photoperiod conditions (16 h light/8 h dark). The leaves of bitter gourd were supported by the Institute of Tropical Crop Genetic Resources in Hainan province, China. The seeds were wrapped in a moist cloth and incubated at 37 °C under dark conditions until germination occurred. Following germination, which typically took 2–3 days, the sprouted seeds were transplanted into small pots on February 15th of 2023. These pots were then placed inside a greenhouse with temperatures ranging from 25 to 28 °C and light intensity of approximately 20,000 lux. The seedlings remained in the greenhouse until they developed 4–6 true leaves. Subsequently, the seedlings were transferred to an open field where they were sown directly into the soil.

The plant DNA was extracted using the Universal Genomic DNA Kit (CWBIO, CW2298M). Primers were designed with a melting temperature (Tm) ranging from 55 to 62 °C and a length of 20–25 base pairs. PCR was performed using the 2×Hieff^®^PCR Master Mix with Dye (Yeasen, 10102ES50). For PCR reaction system (20 ul), about 1 ul (100 ng) DNA, 1 ul primers (F+R, 10 uM/L), 10 ul 2×Hieff^®^PCR Master Mix, 8 ul ddH_2_O. All PCR used the cycling program: initial denature at 95 °C for 5 min; 35 cycles of denature at 95 °C for 30 s, annealing at 55 °C for 30 s, and extension at 72 °C for 30 s; final extension at 72 °C for 5 min. The products were loaded onto a 1% agarose gel and subjected to electrophoresis at a constant voltage of 150 V for 15 minutes following completion of the PCR program.

## Result

**Figure 1:**
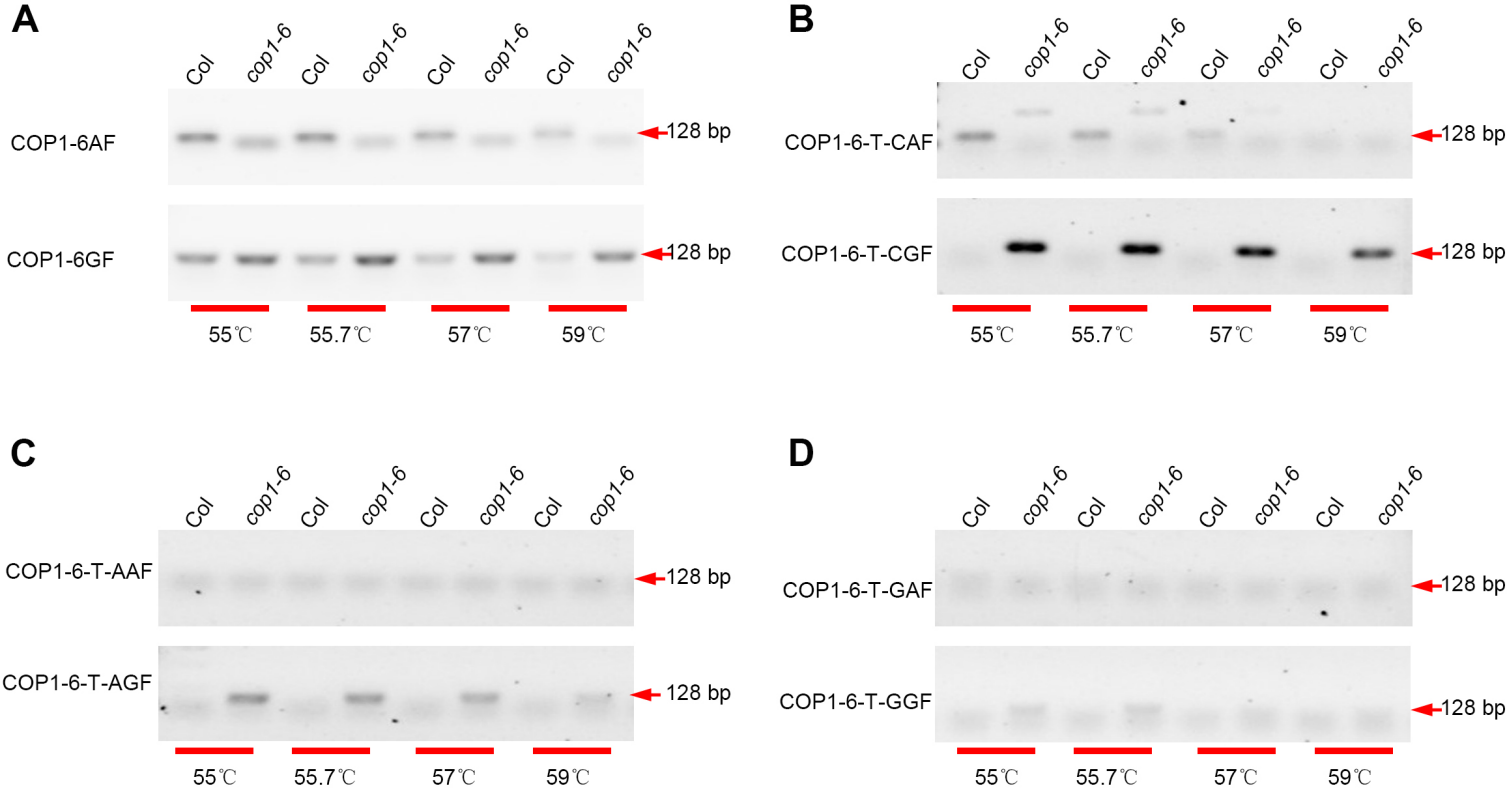
Detection the point mutation between Col and *cop1-6*. Using the DNA of Col and *cop1–6* as template respectively, PCR reactions were performed using the following primer pairs: COP1-6AF/COP1-6SR (upper) and COP1-6GF/COP1-6SR (lower) **(A)**, COP1-6-T-CAF/COP1-6SR (upper) and COP1-6-T-CGF/COP1-6SR (lower) **(B)**, COP1-6-T-AAF/COP1-6SR (upper) and COP1-6-T-AGF/COP1-6SR (lower) **(C)**, COP1-6-T-GAF/COP1-6SR (upper) and COP1-6-T-GGF/COP1-6SR (lower) **(D)**. These reactions were carried out at varying annealing temperatures (55 °C, 55.7 °C, 57 °C, and 59 °C). Subsequently, the PCR products were analyzed via agarose gel electrophoresis.

The *COP1* gene serves as an essential prototypical negative regulator of photomorphogenesis in *Arabidopsis thaliana* (Lau and Deng, 2012; Han et al., 2020), and the mutant *cop1–6* allele possesses a point mutation (A-G) at the 3’ end of intron 4, which results in a 15-bp insertion due to cryptic splicing (Ang and Deng, 1994). To distinguish the *cop1–6* mutant from the wild type (WT), SNP detection is employed to identify the point mutation, with PCR amplification followed by agarose gel electrophoresis (AGE) serving as an efficient method. First, forward and reverse primers were designed, noting that two forward primers differing by 1 bp at the 3’ end were used for the *cop1–6* mutant (COP1-6GF) and WT (COP1-6AF). Second, after PCR amplification, products were analyzed using AGE. Ideally, COP1-6AF/COP1-6SR should amplify only WT, while COP1-6GF/COP1-6SR should amplify only the *cop1–6* mutant. However, both primer pairs amplified both WT and the mutant (**Figure 1A**). To address this issue, a 1 bp mismatch was introduced by replacing the nucleotide T adjacent to the 3’ end with C. The gel image demonstrated that the ‘T-C’ substitution effectively discriminated between the *cop1–6* mutant and WT (**Figure 1B**). When T was replaced with A, only the COP1-6-T-AGF/COP1-6SR primer pair produced visible PCR products (**Figure 1C**), whereas no PCR products were detected with COP1-6-T-GGF/COP1-6SR or COP1-6-T-GAF/COP1-6SR (**Figure 1D**). Additionally, introducing a 1 bp mismatch by replacing the nucleotide G next to the 3’ end did not yield any PCR products (data not shown). These experiments indicate that introducing a ‘T-C’ mismatch at the 3’ end of the forward primer is effective for SNP detection.

**Figure 2:**
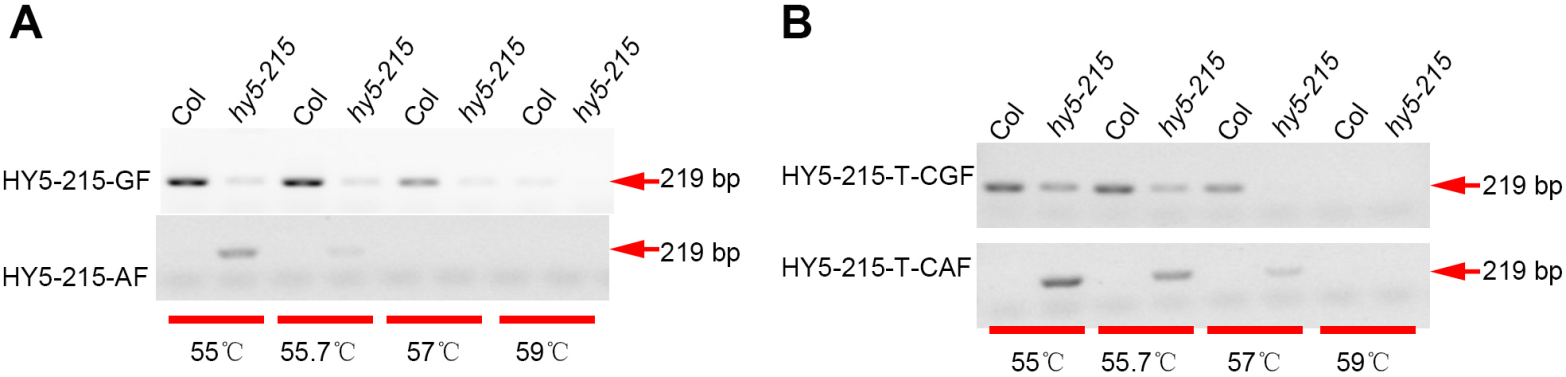
Detection the point mutation between Col and *hy5-215*. PCR reactions were conducted using DNA templates from Col and *hy5-215*, with the following primer pairs employed: HY5-215-GF/HY5-215-R (upper) and HY5-215-AF/HY5-215-R (lower) **(A)**, HY5-215-T-CGF/HY5-215-R (upper) and HY5-215-T-CAF/HY5-215-R (lower) **(B)**. These reactions were carried out at varying annealing temperatures (55 °C, 55.7 °C, 57 °C, and 59 °C). Subsequently, the PCR products were analyzed via agarose gel electrophoresis.

Elongated Hypocotyl 5 (HY5) (Chattopadhyay et al., 1998) serves as a positive regulator in the photomorphogenesis of *Arabidopsis thaliana*. The *hy5–215* mutant harbors a point mutation (G-A) within the first intron. To differentiate the *hy5–215* mutant from the WT, we designed a reverse primer and two forward primers: HY5-215-GF/HY5-215-R for WT, and HY5-215-AF/HY5-215-R for *hy5-215*. AGE analysis revealed that the primer pair HY5-215-GF/HY5-215-R could distinguish the *hy5–215* mutant at an annealing temperature of 57 °C, while HY5-215-AF/HY5-215-R could identify the WT at 55 °C annealing (**Figure 2A**). To further enhance discrimination, we introduced a 1-bp mismatch (‘T-C’) adjacent to the 3’ end of the forward primer. As anticipated, the modified primer pairs HY5-215-T-CGF/HY5-215-R and HY5-215-T-CAF/HY5-215-R successfully discriminated between the *hy5–215* mutant and WT at 57 °C annealing (**Figure 2B**). These findings confirm that a ‘T-C’ mismatch at the 3’ end of the forward primer is effective for single nucleotide polymorphism (SNP) detection.

**Figure 3:**
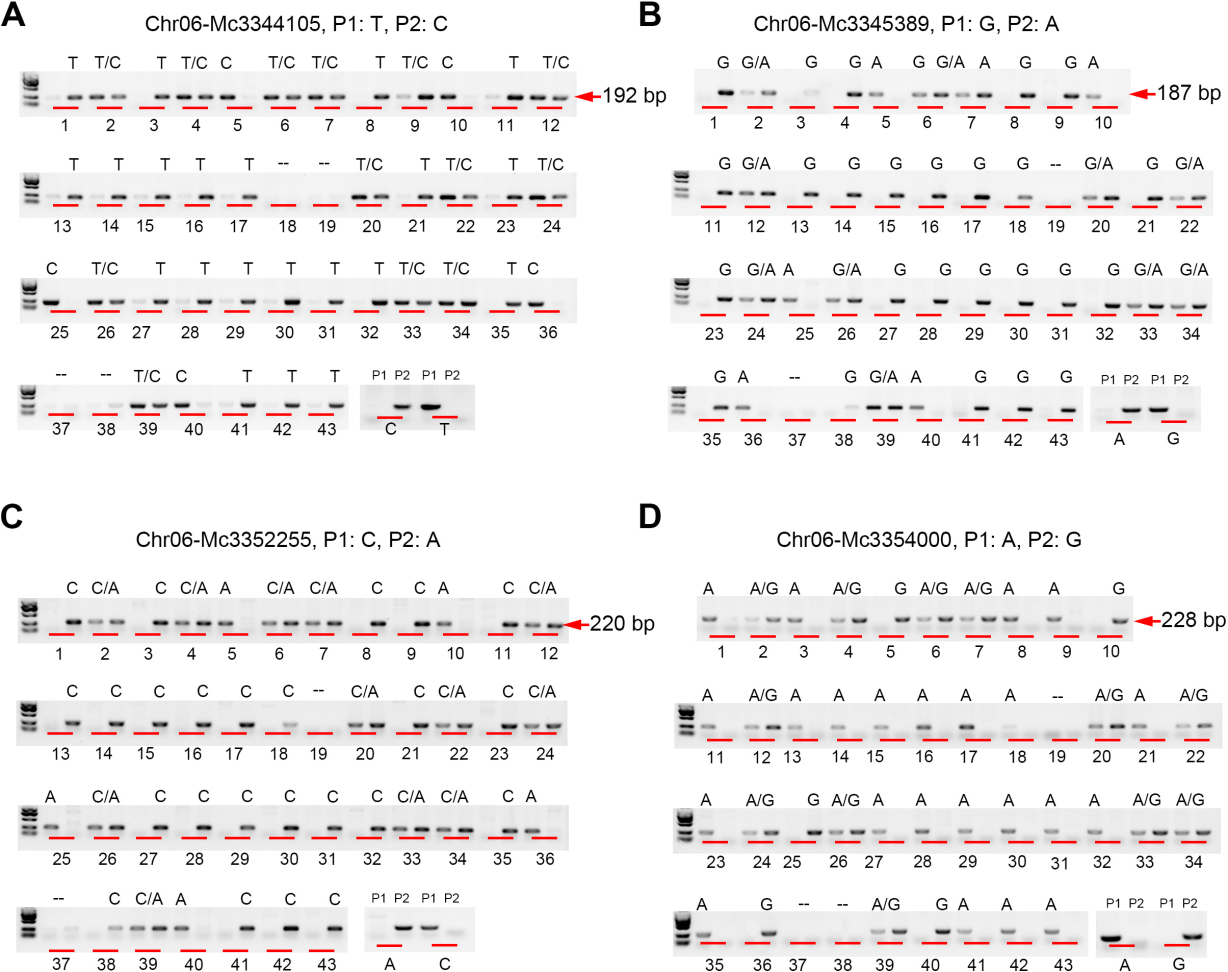
The (‘T-C’) mismatched method was utilized to design SNP markers for detecting the F_2_ generations of bitter gourd. A 1-bp mismatch (‘T-C’) was introduced into the forward primers to detect SNPs at positions Chr06 3,344,105 **(A)**, 3,345,389 **(B)**, 3,352,255 **(C)**, and 3,354,000 **(D)** in bitter gourd. Here, P1 and P2 denote the parental lines, while “–” indicates the absence of PCR products. These reactions were carried out at an annealing temperature of 55 °C. Subsequently, the PCR products were analyzed via agarose gel electrophoresis.

**Figure 4:**
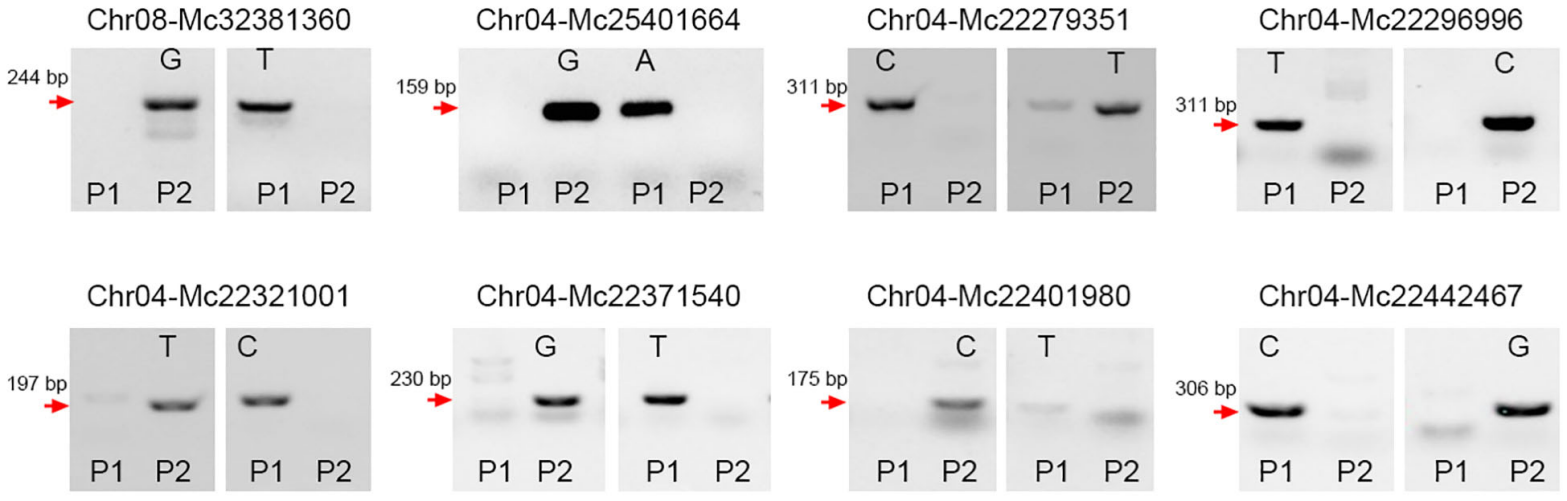
Using the (‘T-C’) mismatched method to design SNP markers in bitter gourd. Eight SNP markers, including Chr08-Mc32381360 (G/T), Chr04-Mc25401664 (G/A), Chr04-Mc22279351 (C/T), Chr04-Mc22296996 (T/C), Chr04-Mc22321001 (T/C), Chr04-Mc22371540 (G/T), Chr04-Mc22401980 (C/T), and Chr04-Mc22442467 (C/G), were developed. P1 and P2 represent two parental lines. These reactions were carried out at an annealing temperature of 55 °C. Subsequently, the PCR products were analyzed via agarose gel electrophoresis.

To expand the application of the SNP detection method, we designed SNP molecular markers for genotyping in bitter gourd (*Momordica charantia*). Fruit length is one of the most important agronomic traits for bitter gourd. Using bulked segregation analysis (BSA), the confidence interval for this trait was localized to chromosome 6 between 909,395 bp and 5,047,012 bp. We selected four candidate SNPs at positions 3,344,105 (C/T), 3,345,389 (A/G), 3,352,255 (A/C), and 3,354,000 (A/G) to evaluate their association with fruit length. By introducing a 1-bp mismatch (‘T-C’) in the forward primers (**Table 1**), we successfully detected these four SNP markers. Furthermore, we validated the effectiveness of this method by genotyping F_2_ populations (**Figures 3A–D**), which confirmed its reliability. Additionally, we employed this method to identify an additional eight SNPs in bitter gourd, specifically: Chr08-Mc32381360 (G/T), Chr04-Mc25401664 (G/A), Chr04-Mc22279351 (C/T), Chr04-Mc22296996 (T/C), Chr04-Mc22321001 (T/C), Chr04-Mc22371540 (G/T), Chr04-Mc22401980 (C/T), and Chr04-Mc22442467 (C/G) (**Figure 4**). With the exception of Chr04-Mc22279351 (C/T) and Chr04-Mc22401980 (C/T), the detection of the other six SNPs performed exceptionally well. For Mc22279351, the primer with a terminal base pair “T” does not clearly distinguish between P1 (gray value: 4510.53) and P2 (16145.90). For Mc22401980, the primer with a terminal base pair “T” exhibits very low amplification efficiency.

**Table 1:** primers used in this study.

Name	Sequence
COP1-6-AF	TTAAAGTGTCTTGTCTTGT**A**
COP1-6-GF	TTAAAGTGTCTTGTCTTGT**G**
COP1S-R	CTATAGCCTTCCCTCCGTAC
COP1-6-T-CAF	TTAAAGTGTCTTGTCTTGc**A**
COP1-6-T-CGF	TTAAAGTGTCTTGTCTTGc**G**
COP1-6-T-AAF	TTAAAGTGTCTTGTCTTGa**A**
COP1-6-T-AGF	TTAAAGTGTCTTGTCTTGa**G**
COP1-6-T-GAF	TTAAAGTGTCTTGTCTTGg**A**
COP1-6-T-GGF	TTAAAGTGTCTTGTCTTGg**G**
COP1-6-G-CAF	TTAAAGTGTCTTGTCTTcT**A**
COP1-6-G-CGF	TTAAAGTGTCTTGTCTTcT**G**
COP1-6-G-TAF	TTAAAGTGTCTTGTCTTtT**A**
COP1-6-G-TGF	TTAAAGTGTCTTGTCTTtT**G**
COP1-6-G-AAF	TTAAAGTGTCTTGTCTTaT**A**
COP1-6-G-AGF	TTAAAGTGTCTTGTCTTaT**G**
HY5-215-GF	GTCTCTTTTATGTTTTAAA**G**
HY5-215-AF	GTCTCTTTTATGTTTTAAA**A**
HY5-215-T-CGF	GTCTCTTTTATGTTTcAAA**G**
HY5-215-T-CAF	GTCTCTTTTATGTTTcAAA**A**
HY5-215-R	TAGAGAAAAAGACACCTCTTCAGCC
Chr06-Mc3344105-CF	TCCGTGAGCAAGGAAAATAGc**C**
Chr06-Mc3344105-TF	TCCGTGAGCAAGGAAAATAGc**T**
Chr06-Mc344105-R	CAAGCTCAAACAAGCATCCA
Chr06-Mc3345389-AF	CTGTTGAGGAAATTAAGcG**A**
Chr06-Mc3345389-GF	CTGTTGAGGAAATTAAGcG**G**
Chr06-Mc3345389-R	ATTCTGAGCCGGAGCAATAA
Chr06-Mc3352255-AF	ATAATTAAGCTTCTTTGCc**A**
Chr06-Mc3352255-CF	ATAATTAAGCTTCTTTGCc**C**
Chr06-Mc3352255-R	TTGTGCTCATATAAGCCCTACATA
Chr06-Mc3354000-AF	AATGCTATCTCAAGTATTc**A**
Chr06-Mc3354000-GF	AATGCTATCTCAAGTATTc**G**
Chr06-Mc3354000-R	TTATCAGTAAAACTCATGCTTTCATTT
Chr08-32381360-GF	CTGTTTGATGCAGATACTcCGC**G**
Chr08-32381360-TF	CTGTTTGATGCAGATACTcCGC**T**
Chr08-32381360-R	AGAGGCTTTGCCTGAAATGA
Chr04-25401664-GF	GGACTTGCCAAATTGTTTTAGGTc**G**
Chr04-25401664-AF	GGACTTGCCAAATTGTTTTAGGTc**A**
Chr04-25401664-R	AGAGCAGCAGACAAGGAACA
Chr04-22279351-CF	GGCGAGCTCGTGGAAGGTc**C**
Chr04-22279351-TF	GGCGAGCTCGTGGAAGGTc**T**
Chr04-22279351-R	TCCAACCATTGATGATCTGC
Chr04-22296996-TF	TGGTTGGGATATCTCTATc**T**
Chr04-22296996-CF	TGGTTGGGATATCTCTATc**C**
Chr04-22296996-R	TGCATGCAAAGAACATTCAAG
Chr04-22321001-TF	CGACAGTGATGAGAACGGc**T**
Chr04-22321001-CF	CGACAGTGATGAGAACGGc**C**
Chr04-22321001-R	GAGGAAAAACGCCATGGATA
Chr04-22371540-GF	ATGTCCTCATATTTGGTGc**G**
Chr04-22371540-TF	ATGTCCTCATATTTGGTGc**T**
Chr04-22371540-R	AGGGAGGGCATGCTTAGACT
Chr04-22401980-CF	AAAAAGCTAAAATAACTCc**C**
Chr04-22401980-TF	AAAAAGCTAAAATAACTCc**T**
Chr04-22401980-R	TGGGATAATTTTTGCCCTCA
Chr04-22442467-CF	TCATCCTGAAAAAAAGAAc**C**
Chr04-22442467-GF	TCATCCTGAAAAAAAGAAc**G**
Chr04-22442467-R	GCCAAAATCACTTTTGTCATTG

The bold nucleotide at the 3’ end of the forward primer indicates either the variant allele or the wild type allele, The lowercased nucleotide indicates a mismatch change that was introduced to the SNP site. F, forward primer; R, reverse primer.

## Discussion

The Polymerase Chain Reaction (PCR) is a highly sensitive and versatile tool widely used in both research and clinical settings for genotyping and the detection of naturally occurring or experimentally induced genetic variations. However, the identification of Single Nucleotide Polymorphisms (SNPs) by PCR remains challenging due to the minimal difference between variant and wild-type alleles—often a single nucleotide substitution. Conventional approaches such as Sanger sequencing and commercial detection kits are typically time-consuming for large-scale SNP analysis. The strategic incorporation of mismatched bases into primer design enables SNP discrimination through PCR amplification followed by agarose gel electrophoresis. Nevertheless, the lack of a standardized protocol for introducing such mismatches presents significant challenges in developing reliable primer sets, often requiring extensive optimization that entails considerable time, labor, and financial resources. This limitation has hindered the broader application of this otherwise promising method. To explore and establish a universal primer design strategy based on targeted base mismatches, a series of primers were developed and evaluated across multiple studies for detecting the *cop1–6* mutant in Arabidopsis thaliana, thereby validating the method’s broad applicability. During genetic screening experiments aimed at distinguishing the wild type from the *cop1–6* mutant, we observed that substitutions of A, G, or C within 2–3 base pairs upstream of the 3′ end of the forward primer resulted in unstable amplification outcomes, including failed amplification or non-specific amplification of both alleles. Stable and specific discrimination was achieved only when T was substituted with C, generating an A–C mismatch. Substitutions with A or G at this position led to reduced amplification efficiency or inadequate differentiation between genotypes. This optimized approach represents a significant improvement over existing methods, such as those employing mismatches at the −2 position without specifying the most effective mismatching base pair (Chen and Schedl, 2021).

In this study, difficulties were encountered in designing primers to discriminate the wild-type negative regulator *AtDET1* and its mutant *det1-1* (G/A) (Pepper and Chory, 1997), as well as the SNP locus Chr04-Mc22357388 (C/T) in bitter gourd. The inability to clearly differentiate the *det1–1* mutant from the wild type in Arabidopsis thaliana is likely attributable to the excessively high GC content (74%) in the forward primer. In contrast, the GC content at the corresponding region of the bitter gourd SNP site Chr04-Mc22357388 (C/T) was only 16%, which falls outside the optimal range. Across this study, 14 single nucleotide polymorphisms (SNPs) were successfully identified, and the GC content of the designed forward primers ranged from 21% to 63%. To ensure optimal primer design for comprehensive SNP detection, it is recommended that the GC content of forward primers fall within the range of 21% to 63%. In conclusion, the SNP detection strategy described in this study is not only facile and time-efficient but also exhibits remarkable flexibility and cost-effectiveness, offering practical advantages for routine genotyping applications.

## Data Availability

The original contributions presented in the study are included in the article/supplementary material. Further inquiries can be directed to the corresponding authors.
